# Positivity Status and Molecular Characterization of Porcine Parvoviruses 1 Through 8 (PPV1-PPV8) from Slaughtered Pigs in China

**DOI:** 10.3390/ani14223238

**Published:** 2024-11-12

**Authors:** Dashi Zhao, Hong Lin, Zitao Huang, Yajie Zhou, Wenhao Qi, Meng Cui, Ming Qiu, Jianzhong Zhu, Nanhua Chen

**Affiliations:** 1 College of Veterinary Medicine, Yangzhou University, Yangzhou 225009, China; 18246986065@163.com (D.Z.); linhonglynn@yzu.edu.cn (H.L.); zhouyj202405@163.com (Y.Z.); qiwenhao2829@163.com (W.Q.); cuimeng201902@163.com (M.C.); qiuming1997@126.com (M.Q.); jzzhu@yzu.edu.cn (J.Z.); 2 Joint International Research Laboratory of Agriculture and Agri-Product Safety, Yangzhou 225009, China; 3 International Research Laboratory of Prevention and Control of Important Animal Infectious Diseases and Zoonotic Diseases of Jiangsu Higher Education Institutions, Yangzhou 225009, China; 4 Jiangsu Co-Innovation Center for Prevention and Control of Important Animal Infectious Diseases and Zoonoses, Yangzhou University, Yangzhou 225009, China; 5 Comparative Medicine Research Institute, Yangzhou University, Yangzhou 225009, China; 6 Animal Health Supervision Institute of Fengxi District, Chaozhou 521031, China; 13450818935@163.com

**Keywords:** PPV1-PPV8, prevalence, co-infection, genome, slaughtered pigs

## Abstract

Porcine parvoviruses one through eight (PPV1-PPV8) are prevalent in Chinese swine herds. However, the infection status of all these PPVs in slaughtered pigs is still unclarified. In this study, we detected PPV1-PPV8 in 353 tissue samples collected from slaughtered pigs from six regions of China in 2023. At least one species of PPV was detected in 79.32% of the samples (280 out of 353). Six PPV species were detected, except for PPV4 and PPV8, in slaughtered pigs, within which PPV3 (49.86%), PPV2 (42.49%), and PPV7 (42.21%) were predominant, followed by PPV1 (13.31%), PPV6 (13.31%), and PPV5 (8.22%). Noticeably, co-infection was frequently detected, with 67.50% of PPV-positive samples (189 out of 280) co-infecting with two to six PPVs. In addition, one representative genome for each detected PPV was determined. Multiple sequence alignment determined a large number of substitutions in capsid proteins of PPVs. Genome-based phylogenetic analysis confirmed the PCR detection results. Recombination detection identified two potential recombinants (PPV2 GDCZ2023-2088 strain and PPV3 HLJSYS2023-1654 strain) in slaughtered pigs. Overall, this study provides new insights into the prevalence and evolution of PPVs, particularly in slaughtered pigs in China.

## Simple Summary

In addition to porcine parvovirus 1 (PPV1), seven new PPVs (PPV2-PPV8) have been identified in the last two decades. However, the prevalence and evolution of PPVs in slaughtered pigs in China are still unclear. The infection status in slaughtered pigs could reflect the overall health situation during pig production in swine herds. Therefore, we detected the infection status of PPVs in 353 samples collected from slaughtered pigs in six regions of China in 2023. Overall, 79.32% of the samples were PPV positive, with 67.50% PPV-positive samples co-infecting with two to six PPVs. Six species of PPVs were detected, except for PPV4 and PPV8. Representative PPV genomes were determined for evaluating evolutionary relationships and detecting recombination events. A genome-based phylogenetic tree confirmed the PCR results, and cross-over events were detected in the PPV2 and PPV3 strains identified in this study. This study provided the first clue on the prevalence and evolution of PPVs in slaughtered pigs in China.

## 1. Introduction

Parvoviruses (PVs) are small non-enveloped DNA viruses containing a linear single-stranded genome, which may infect a wide range of animals [1]. In mammals, PVs are closely associated with nervous and respiratory diseases in humans [2], hepatitis in horses [3], enteritis in dogs [4], panleukopenia in cats [5], and reproductive diseases in pigs [6]. The genomes of porcine parvoviruses (PPVs) are about 4.0 ~ 6.3 kb in length, containing two open reading frames (ORF1 and ORF2). ORF1 encodes a nonstructural protein, whilst ORF2 encodes a capsid protein [7]. Eight PPVs (PPV1 to PPV8) have been detected in wild boars and domestic pig herds [8,9,10], which can be divided into four genera of parvoviruses [7,11]. In detail, PPV1 and PPV8 belong to *Protoparvovirus*, PPV2 and PPV3 are grouped in *Tetraparvovirus*, PPV4, PPV5, and PPV6 are clustered within *Copiparvovirus*, and PPV7 is grouped within *Chaphamaparvovirus*. PPV1 was first isolated in Germany in 1965 [12]. PPV2 was unexpectedly amplified from swine sera in Myanmar in 2001 [13]. PPV3 (porcine hokovirus) was first identified in Hong Kong in 2008 [14]. PPV4 was detected in a diseased pig co-infected with PCV2 in the United States in 2010 [15]. PPV5 was identified in the United States in 2013 [16]. PPV6 was found in aborted pig fetuses in China in 2014 [17]. PPV7 was first described in healthy adult pigs in the United States in 2016 [18]. PPV8 was identified in PRRSV-positive samples in China in 2021 [11]. PPVs are widely spread in wild boars all around the world, including Serbia, Italy, Spain, Turkey, Romania, South Korea, and China [7,9,10,19]. They are generally detected as the most common pathogens in wild boars. For instance, two recent studies showed that PPVs could be detected in 56% and 44.4% of samples from Serbia and Italy, respectively [9,10]. More importantly, all PPVs could be detected from clinically healthy and diseased domestic pigs. The clinical symptoms may include fever, rash, dyspnea, porcine dermatitis, and nephropathy syndrome (PDNS), while the potential pathological lesions include lung consolidation, lymph node hemorrhage, kidney pathology, liver pathology, pericardium pathology, enteric pathology, meningitis, and lymphadenopathy [7]. PPV1 is the major agent causing SMEDI syndrome (stillbirths, mummification, embryonic death, and infertility) [7]. Even though the pathogenicity of new PPVs (PPV2-PPV8) is still unclarified, they have been proposed as potential pathogens of porcine respiratory disease complex (PRDC) [20]. Although there are PPV commercial vaccines available, PPV infection is still not well controlled worldwide. Therefore, it is important to monitor the prevalence and evolution of PPVs in pig populations.

PPV1 was first identified in China in 1983 [21]. In the last two decades, all new PPVs (PPV2 to PPV8) have also been detected in Chinese swine herds [7,11,14,17]. In addition, previous studies showed that PPVs can be detected at every stage of the pig production cycle in different types of samples (such as lung and lymph nodes) [7,22,23]. A recent study detected PPVs in the gilts showing that new PPVs (such as PPV4 and PPV6) might affect reproductive performance [23]. Noticeably, PPVs are more prevalent in finishing and nursery pigs than in suckling pigs [7]. The infection status in slaughtered pigs could reflect the overall health situation during pig production in swine herds. However, the infection status of PPVs in slaughtered pigs has not been determined yet. Moreover, mutation and recombination are two key mechanisms for virus evolution. Mutations in PPVs are potentially associated with variations in the pathogenicity and protective immune responses [24,25]. Recombination events are also frequently detected in different PPVs, including PPV1, PPV2, PPV3, and PPV7 [7,26]. However, the mutation and recombination situations of PPVs in slaughtered pigs were still unknown. In addition, PPVs have been detected in different types of tissues, indicating that they have broad tropism [27]. However, they were more commonly found in lung and lymphoid tissues such as lymph nodes, tonsils, and spleens [8], Therefore, we evaluated the infection status of PPVs in slaughtered pigs using 353 tissue samples (lungs and lymph nodes) collected from six regions of China in 2023. Furthermore, representative PPV-positive samples were submitted to complete genome sequencing, multiple sequence alignment, genome-based phylogenetic analysis, and recombination detection.

## 2. Materials and Methods

### 2.1. Sample Collection

A total of 353 tissue samples (including 338 lungs and 15 lymph nodes) from slaughtered pigs were submitted from six regions (Heilongjiang, Shandong, Sichuan, Henan, Guangdong, and Beijing) of China to Yangzhou University from February 18th to December 10th in 2023. These samples were mailed from slaughterhouses from distinct regions of China. However, detailed information about these slaughterhouses or animal owners was not provided. Therefore, we only record the regions where the samples came from. Even though neither the slaughterhouse owners nor the animal owners provided the hard copy consents to give us the permissions of sample collection, they, by default, allowed us to use these samples by submitting them to our laboratory for potential viral detection.

### 2.2. PPV Detection

The infection status of PPVs (PPV1-PPV8) in slaughtered pigs was determined by PCR assays described previously [7,11]. Briefly, total DNAs were extracted from tissue samples using the HiPure Tissue DNA minikit (Magen, Guangzhou, China). Viral DNAs were eluted using 50 μL nuclease-free double distilled water (ddH_2_O) and stored at −40 °C until used. The concentrations of primer pairs and amplification conditions were optimized accordingly [7]. Each of the PCR assays was performed in a 20 μL reaction system containing 2 μL DNA, 0.5 μL corresponding primer pair (10 μM), 7.5 μL ddH_2_O, and 10 μL Premix Taq (TaKaRa, Dalian, China). The amplification was carried out at 35 cycles of 98 °C 10 s, 55 °C 30 s, and 72 °C for 1 min according to the manufacturer’s instructions. The PCR products were detected in 1.0% agarose gel electrophoresis with a 1× TAE buffer. The obtained amplicons were sent out for Sanger sequencing to confirm the PCR results (Genewiz, Suzhou, China).

### 2.3. PPV Genome Sequencing

Representative PPV-positive samples were used for complete genome sequencing with primers shown in our previous study [7]. The primers could amplify overlapped fragments covering the entire PPV genome. Each fragment was triply sequenced to ensure the accuracy of each sequence. The obtained sequences were assembled using DNAMAN 6.0 software, and six nearly complete PPV genomes were obtained in this study.

### 2.4. Multiple Alignment and Phylogenetic Analysis

To estimate the evolutionary relationships between PPV genomes obtained from slaughtered pigs in this study and other PPV genomes from the GenBank database, the obtained six PPV genomes and forty representative PPV genomes (5 for each PPV) were aligned by the ClustalX 2.1 (University College Dublin, Dublin, Ireland) [28,29]. And, then, a genome-based phylogenetic tree was constructed with MEGA 6.06 (Tokyo Metropolitan University, Tokyo, Japan) [7,30]. The phylogenetic tree was built using the 46 aligned sequences by the neighbor-joining method and the maximum composite likelihood model, including transitions and transversions, substitutions, homogeneous patterns among lineages, and uniform rates among sites. In addition, the complete deletion option to treat gaps and missing data were selected. The robustness was estimated by bootstrapping with 1000 replicates.

### 2.5. Recombination Detection

To determine the intraspecies recombination events during the generation of PPV strains from slaughtered pigs, all available PPV genomes (45 PPV1, 121 PPV2, 49 PPV3, 79 PPV5, 105 PPV6, and 145 PPV7), except for PPV4 and PPV8, from the GenBank database were used for screening the cross-over events by recombination detection program 4 (RDP4) (University of Cape Town, Cape Town, South Africa) [31]. Seven methods, including RDP, GENECONV, BootScan, MaxChi, Chimaera, SiScan, and 3Seq, were utilized for recombination detection and breakpoint determination. The *p* value cut off was set at 0.05. *p* < 0.05 indicated that the cross-over events are significant.

## 3. Results

### 3.1. The Prevalence of PPVs in Slaughtered Pigs in China

To test the prevalence of PPVs in slaughtered pigs in 2023, 353 tissue samples collected from six regions of China were detected. As shown in Table 1 and Table S1, at least one species of PPV was detected in 79.32% of the samples (280 out of 353). Six PPVs (PPV1, PPV2, PPV3, PPV5, PPV6, and PPV7) were detected, except for PPV4 and PPV8, in slaughtered pigs, within which PPV3 (176 out of 353, 49.86%), PPV2 (150/353, 42.49%), and PPV7 (149/353, 42.21%) were predominant, followed by PPV1 (47/353, 13.31%), PPV6 (47/353, 13.31%), and PPV5 (29/353, 8.22%). In addition, all six PPVs could be detected in both lung and lymph node samples (Table S1). These results showed that PPVs are highly prevalent in slaughtered pigs in China.

### 3.2. Co-Infection Status of PPVs in Slaughtered Pigs

To clarify the co-infection status of PPVs in slaughtered pigs, the positivity of each sample was determined. As shown in Table 2, simplex, duplex, triplex, quadruplex, quintuplex, and sextuplex PPV infections were detected in 91, 102, 59, 17, 8, and 3 tissue samples from slaughtered pigs. In PPV-positive samples, even though 32.50% of the samples (91 out of 280) were infected by one species of PPV, co-infection was frequently detected, with 67.50% of the samples (189 out of 280) co-infecting with two to six types of PPVs. In addition, similar co-infection status of PPVs could be detected in both lung and lymph node samples (Table S1). These results supported that co-infection among different species of PPVs commonly occurred in slaughtered pigs in China.

### 3.3. Evolutionary Relationship Evaluation

**Figure 1:**
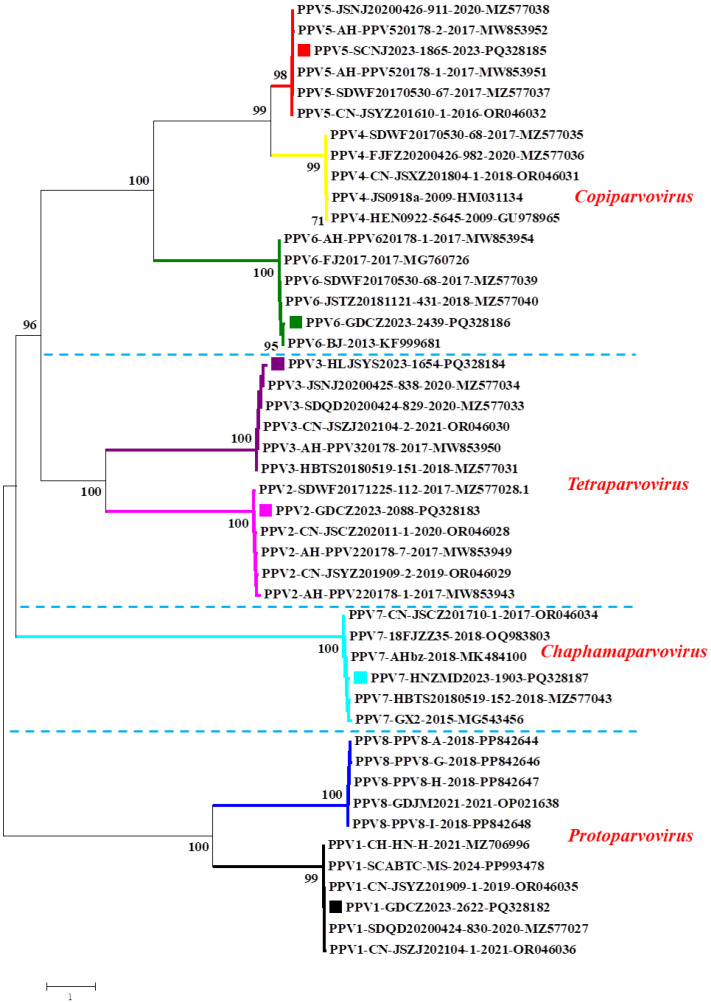
Genome-based phylogenetic analysis. The phylogenetic tree was constructed based on six PPV genomes obtained in this study and forty representative PPV genomes (five for each PPV) from GenBank. Distinct PPVs are shown in different colors. PPVs are clustered within four genera. Our PPV strains are highlighted with colored squares. Each virus is presented by species, virus name, year of identification, and GenBank accession number. Bootstrap values from 1000 replications are shown in each node.

To estimate the molecular characteristics and evolutionary relationships between PPVs identified in this study and other Chinese PPVs, six PPV genomes (one for each species of PPV detected positive in this study) from slaughtered pigs were determined, as shown previously [7]. The obtained nearly complete PPV genomes were deposited into the GenBank database with accession numbers PQ328182-PQ328187 (Table 3). The blast results showed that our PPV genomes shared high similarity with other PPV genomes in the GenBank database. The PPV1 GDCZ2023-2622 strain shared the highest homology (99.81%) with the PPV1KUIP22-4 isolate (GenBank No. OP377056) and >99.00% similarity with other PPV1 strains. The PPV2 GDCZ2023-2088 strain showed the highest homology (99.75%) with the HuB21-2016 isolate (MN326157) and >96.66% similarity with other PPV2 genomes. The PPV3 HLJSYS2023-1654 strain shared the highest homology (98.48%) with the HBTS20180519-151 strain (MZ577031) and >96.89% similarity with other PPV3 strains. The PPV5 SCNJ2023-1865 strain showed the highest homology (99.70%) with the SDWF20170530-67 strain (MZ577037) and >99.18% similarity with other PPV5 genomes. The PPV6 GDCZ2023-2439 strain shared the highest homology (98.37%) with the SC strain (KF999684) and >97.81% similarity with other PPV6 strains. The PPV7 HNZMD2023-1903 strain showed the highest homology (95.43%) with the GX49 strain (NC_040562) and >93.35% similarity with other PPV7 genomes. The genome-based phylogenetic tree showed that our PPV genomes obtained in this study were grouped together with corresponding PPV genomes (Figure 1). The phylogenetic results not only confirmed the accuracy of the PCR results but also supported the close evolutionary relationship between PPVs in slaughtered pigs and PPVs from other production stages of pigs.

### 3.4. Recombination Events and Substitutions

**Figure 2:**
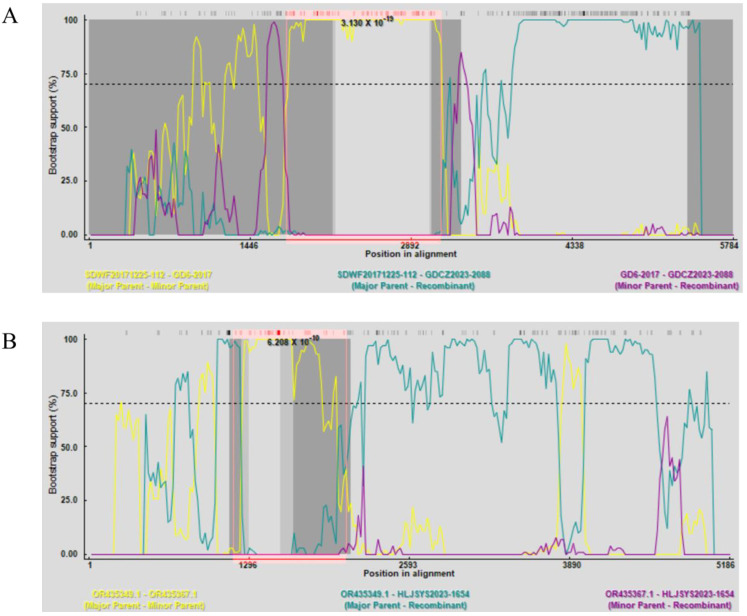
Potential recombination events detected in our PPV2 and PPV3 strains by RDP4. (**A**) The PPV2 GDCZ2023-2088 strain was recombined from the major parental virus SDWF20171225-112 and the minor parental virus GD6-2017. (**B**) The PPV3 HLJSYS2023-1654 strain was recombined from parental GD202206-4 and SD202203-3 viruses. The *p* value identified by the BootScan method for each cross-over event was also shown.

To evaluate the role of recombination in the generation of PPVs from slaughtered pigs identified in this study, we aligned our PPV genomes with all available corresponding PPV genomes in GenBank and submitted them to intraspecies recombination analyses. PPV2 GDCZ2023-2088 and PPV3 HLJSYS2023-1654 strains were detected as recombinants by all seven methods in RDP4 (Table 4). The PPV2 GDCZ2023-2088 strain was recombined from SDWF20171225-112 and GD6-2017 viruses (Figure 2A), while PPV3 HLJSYS2023-1654 was generated by a recombination event between GD202206-4 and SD202203-3 viruses (Figure 2B). In addition, the alignments of PPV capsid proteins identified a large number of substitutions in our PPVs when compared with other Chinese PPVs (Figure S1). These results supported that finishing pigs (slaughtered pigs) could also serve as an important host for the evolution of PPVs in China.

## 4. Discussion

All PPVs have been widely spread in Chinese swine herds. The majority of previous studies explored the infection status of one specific PPV or different PPVs in a specific region or specific farms [11,14,17,21,32,33,34]. However, very few studies focused on the prevalence and evolution of PPVs in a specific stage of the pig production cycle. In this study, we evaluated the infection status and evolution of PPVs in slaughtered pigs in China in 2023. Considering that PPVs have broad tissue tropism, they have been detected in a large number of tissue samples, including the lung, liver, brain, kidney, spleen, heart, thymus, and thyroid [27]. PPV1 has a tropism to macrophages that could migrate to the placenta and then infect the fetus [35]. PPV2-PPV8 can be detected in serum and tissues, mainly in lung and lymphoid tissues [8]. Therefore, lung and lymph node samples were collected and used for PPV detection in this study. Our results showed that several PPVs are highly prevalent in slaughtered pigs in China. In addition, mutation and recombination analyses showed that finishing pigs (slaughtered pigs) may also serve as a critical host for PPV evolution.

The prevalence of distinct PPVs in China was extensively studied. The percentages of PPV1- to PPV8-positive samples might range from 0 to 75% [11,17,32,36,37,38]. However, the infection status of PPVs in a specific pig production stage was rarely evaluated. Replacement gilts play an essential role in the reproductive cycle. PPV infection in gilts might affect reproductive performance. A recent study on the gilts showed that PPVs are highly prevalent in gilts, with PPV3 (40.1%), PPV5 (20.5%), PPV6 (17%), and PPV1 (14.5%) serving as prevalent viruses [23]. PPV infection in slaughtered pigs could reflect the overall PPV infection status in swine herds. Our results showed that PPVs are also highly prevalent in slaughtered pigs in China, with PPV3 (49.86%), PPV2 (42.49%), and PPV7 (42.21%) being the predominant viruses. Even though PPV4 and PPV8 were not detected in our tissue samples from slaughtered pigs in 2023, the exact infection statuses of PPV4 and PPV8 in slaughtered pigs required further investigation using more spatiotemporal representative samples.

The co-infections among distinct PPVs and PPVs with other pathogens, such as PCV2 and PRRSV, commonly occurred [7,8,23]. Viral pathogens, such as PPV1, PCV2, and PRRSV, and bacterial pathogens, including *Mycoplasma hyopneumoniae,* are all major pathogens for porcine reproductive failure and PRDC [8]. Even though the specific role of each pathogen in PRDC is still unclear, it is well known that co-infection of PPVs with PCV2 and PRRSV could influence the severity of clinical diseases [7]. In addition, new PPVs, such as PPV2, have also been associated with PRDC [20]. Therefore, not only the co-infection of PPVs with other pathogens but also the co-infection among distinct PPVs might play synergistic roles in PPV pathogenicity. In this study, co-infections among PPV1, PPV2, PPV3, PPV5, PPV6, and PPV7 were frequently detected in slaughtered pigs. However, due to the unknown healthy condition of these slaughtered pigs and no cell lines that could be used to isolate new PPVs, it is still a huge challenge to explore the influence of PPV infection and co-infection on the pathogenicity. In light of the complexity of co-infections among distinct pathogens in pigs, overall biosafety prevention and control strategies in a farm level rather than individual pig or individual disease treatments must be preferential.

To evaluate the evolutionary relationships between Chinese PPVs detected in slaughtered pigs and other Chinese PPVs, six representative PPV genomes were determined and submitted to genome-based phylogenetic analysis. Our PPVs shared high genomic similarities (>93.35%) with corresponding PPVs and had close evolutionary relationships with corresponding PPVs, suggesting that there was no significant genetic difference between PPVs in slaughtered pigs and PPVs in other stages of the pig production cycle.

Mutation is one of the major mechanisms for viral evolution. Five mutations (I215T, D378G, H383Q, S436P, and R565K) in the capsid protein of the PPV1 Kresse strain are potentially associated with the pathogenicity [24]. Substitutions at 378, 383, and 565 residues in the 3-fold spike region might also influence immune response [25,39]. Antisera from pigs infected by various PPV1 strains had high neutralizing activities against homologous PPV1 strains but low neutralizing activities against heterologous viruses [25]. A large number of substitutions were identified in our PPVs from slaughtered pigs. Whether these mutated PPVs would change their pathogenicity or affect protective immune responses required further investigation.

In addition to mutation, recombination also plays a critical role in the generation of viral genomic diversity. Recombination events are frequently detected in parvoviruses, including interspecies recombination in rodent parvoviruses and intraspecies recombination in porcine parvoviruses [26]. Intraspecies recombination events have been detected in PPV1, PPV2, PPV3, and PPV7, while no cross-over events were reported in PPV4, PPV5, PPV6, and PPV8 [7,8,26]. PPV1 2074-7 and 225b isolates were detected as potential recombinants [7,8,40]. Strong recombination signals were detected in nine PPV2 strains (F3-12R, F7-1BV, WB-102R2, WB-826MR, WB-763S, WB-804D, WB-720I, F1-23M, and F4-44M) and three PPV3 strains (F2-47M, WB-RO-369, and WB-RO-834) both in domestic pigs and wild boars [41]. Two PPV7 strains (KF4 from South Korea and HBTZ20180519-152 from China) were detected as recombinants originating from wild boars [7]. In this study, the PPV2 GDCZ2023-2088 recombinant was recombined from two PPV2 strains from domestic pigs, while the PPV3 HLJSYS2023-1654 recombinant was generated by two PPV3 strains with unclarified origin. Both this study and previous reports confirmed that PPV recombination events could be detected within and between PPVs from the ages of pigs, within and between regions/countries, and within and between strains originating from domestic pigs and wild boars [8,37]. Overall, both the mutations and recombination events identified in our PPVs from slaughtered pigs confirmed that PPVs keep evolving in finishing pigs (slaughtered pigs) in China.

This is the first study to focus on the prevalence and evolution of PPVs in slaughtered pigs in China. The results from this study supported that PPVs persistently infect pigs, even at the late stage of the pig’s production cycle. In addition, the evolution of PPVs would not stop, even when the host pig was slaughtered. These findings emphasize the importance of biosecurity during the entire pig production cycle. However, only a small number of tissue samples were detected in this study. In addition, these samples were only collected from six regions of China within one year (2023). Due to these limitations, the results from this study might not reflect the exact infection status of PPVs in slaughtered pigs in China. Therefore, more spatially and temporally representative samples should be collected and evaluated in the near future.

## 5. Conclusions

This study provided the first clue on the infection status of PPVs in slaughtered pigs in China. In addition, our results also confirmed that finishing pigs (slaughtered pigs) also serve as a non-negligible host for PPV evolution.

## Figures and Tables

**Table 1:** Distribution of PPV1–8 in 353 samples from slaughtered pigs from different regions of China in 2023.

Region	No.	PPV1	PPV2	PPV3	PPV4	PPV5	PPV6	PPV7	PPV8
Heilongjiang	31 *	+	+	+	-	-	+	+	-
Shandong	37	+	+	+	-	+	+	+	-
Sichuan	34	-	+	+	-	+	+	+	-
Henan	70	+	+	+	-	+	+	+	-
Guangdong	167	+	+	+	-	+	+	+	-
Beijing	14	+	+	+	-	+	+	+	-
Total	353	47/353 (13.31%)	150/353 (42.49%)	176/353 (49.86%)	0/353 (0%)	29/353 (8.22%)	47/353 (13.31%)	149/353 (42.21%)	0/353 (0%)

* The number indicates sample numbers collected from each region.

**Table 2:** Infection and co-infection of distinct PPVs in clinical samples.

Infection Status	Numbers	Types
Simplex infection	91	PPV1 (11) *, PPV2 (16), PPV3 (28), PPV5 (1), PPV6 (2), PPV7 (33)
Duplex infection	102	PPV1+2 (3), PPV1+3 (9), PPV1+6 (1), PPV1+7 (3), PPV2+3 (35), PPV2+5 (2), PPV2+6 (1), PPV2+7 (16), PPV3+6 (6), PPV3+7 (19), PPV5+6 (1), PPV5+7 (4),PPV6+7 (2)
Triplex infection	59	PPV1+2+3 (4), PPV1+2+7 (2), PPV1+3+5 (1), PPV1+3+6 (1), PPV1+3+7 (1), PPV1+5+7 (1), PPV2+3+5 (2), PPV2+3+6 (4), PPV2+3+7 (33), PPV2+5+6 (1), PPV2+5+7 (2), PPV2+6+7 (2), PPV3+6+7 (5)
Quadruplex infection	17	PPV1+2+3+7 (3), PPV2+3+5+6 (2), PPV2+3+5+7 (4), PPV2+3+6+7 (7), PPV3+5+6+7 (1)
Quintuplex infection	8	PPV1+2+3+6+7 (4), PPV2+3+5+6+7 (4)
Sextuplex infection	3	PPV1+2+3+5+6+7 (3)
Septuplex infection	0	/ ^#^
Octuplex infection	0	/

* The numbers in the brackets are the sample numbers of each type of infection status. ^#^ The diagonal indicates none.

**Table 3:** Six PPV genomes from slaughtered pigs determined in this study.

Species	Name	Region *	Collection time	GenBank No.
PPV1	GDCZ2023-2622	Chaozhou, Guangdong	11 November 2023	PQ328182
PPV2	GDCZ2023-2088	Chaozhou, Guangdong	23 April 2023	PQ328183
PPV3	HLJSYS2023-1654	Shuangyashan, Heilongjiang	27 February 2023	PQ328184
PPV5	SCNJ2023-1865	Neijiang, Sichuan	1 March 2023	PQ328185
PPV6	GDCZ2023-2439	Chaozhou, Guangdong	10 September 2023	PQ328186
PPV7	HNZMD2023-1903	Zhumadian, Henan	2 March 2023	PQ328187

* The region indicated the city and province from where the sample was collected.

**Table 4:** Cross-over events identified by RDP4 in this study.

Species		PPV2	PPV3
Recombinant Virus		GDCZ2023-2088	HLJSYS2023-1654
Parental viruses	Major	SDWF20171225-112	GD202206-4
	Minor	GD6-2017	SD202203-3
Breakpoints ^a^	Begin	1669	1167
	End	3052	2083
Score for the seven detection methods embedded in RDP4 ^b^	RDP	2.3 × 10^−4^	6.2 × 10^−10^
	GENECONV	4.0 × 10^−11^	3.2 × 10^−7^
	BootScan	2.5 × 10^−16^	6.2 × 10^−10^
	MaxChi	1.2 × 10^−5^	8.8 × 10^−9^
	Chimaera	6.4 × 10^−6^	6.2 × 10^−7^
	SiScan	1.6 × 10^−11^	8.4 × 10^−22^
	3Seq	1.9 × 10^−11^	7.2 × 10^−10^

^a^ The breakpoints are based on the locations in the genome of the PPV2 GDCZ2023-2088 and PPV3 HLJSYS2023-1654 strains. ^b^ The *p* value cut off is set at 0.05. *p* < 0.05 indicates that the cross-over events are significant.

## Data Availability

The obtained nearly complete PPV genomes have been submitted to GenBank with accession numbers PQ328182-PQ328187.

## Supplementary Materials

The following supporting information can be downloaded at https://www.mdpi.com/article/10.3390/ani14223238/s1, Table S1. Clinical sample infection status. The detailed infection and co-infection information of PPVs in 353 tissue samples collected from six regions of China in 2023 are shown. Figure S1. Multiple alignments of capsid proteins. (A–F) The capsid proteins from our PPVs were compared with capsid proteins from five corresponding representative PPVs.
