# Supplementary material for: Positivity Status and Molecular Characterization of Porcine Parvoviruses 1 Through 8 (PPV1-PPV8) from Slaughtered Pigs in China
Source: Animals (Basel). 2024 Nov 12;14(22):3238. doi: 10.3390/ani14223238 (PMC11591526; doi:10.3390/ani14223238)
Supplement: Supplementary file 1 [file animals-14-03238-s001.zip › Figure S1 Multiple alignments of capsid proteins.pdf]

A: PPV1 capsid protein alignment

```
GDCZ2023-2622     MSENVEQHNPINAGTELSATGNESGGGGGGGGGGRGAGGVGVSTGSFNNQTEFQYLGEGLVRITAHASRLIHLNMPEHETYKRIHVLNSESGVAGQMVQDDAHTQMVTPWSLIDANAWGVWFNPADWQLISNNMTEINLVSFEQEIFNVVL 150
JSYZ201909-1      ...................................................................................................................................................... 150
JSZJ202104-1      ...................................................................................................................................................... 150
SDQD20200424-830  ...............................................................................................................................................A...... 150
CH-HN-H           ...................................................................................................................................................... 150
SCABTC-MS         ...................................................................................................................................................... 150

GDCZ2023-2622     KTITESATSPPTKIYNNDLTASLMVALDTNNTLPYTPAAPRSETLGFYPWLPTKPTQYRYYLSCTRNLNPPTYTGQSQQITDSIQTGLHSDIMFYTIENAVPIHLLRTGDEFSTGIYHFDTKPLKLTHSWQTNRSLGLPPKLLTEPTTEG 300
JSYZ201909-1      ..................................................................................T................................................................... 300
JSZJ202104-1      ..................................................................................T................................................................... 300
SDQD20200424-830  ................................................................I..................................................................................... 300
CH-HN-H           .............................................................................E........................................................................ 300
SCABTC-MS         .............................................................................E........................................................................ 300

GDCZ2023-2622     DQHPGTLPAANTRKGYHQTINNSYTEATAIRPAQVGYNTPYMNFEYSNGGPFLTPIVPTADTQYNDDEPNGAIRFTMGYQHGQLTTSSQELERYTFNPQSKCGRAPKQQFNQQAPLNLENTNNGTLLPSDPIGGKPNMHFMNTLNTYGPL 450
JSYZ201909-1      ...................................................................................................................................................... 450
JSZJ202104-1      ...T...............T.............................................N........................................N........................................... 450
SDQD20200424-830  ...................T...............................................................................................................S.............. 450
CH-HN-H           .................................................................................................................S....Q................T.............. 450
SCABTC-MS         .................................................................................................................S....Q................T.............. 450

GDCZ2023-2622     TALNNTAPVFPNGQIWDKELDTDLKPRLHVTAPFVCKNNPPGQLFVKIAPNLTDDFNADSPQQPRIITYSNFWWKGTLTFTAKMRSSNMWNPIQQHTTTAENIGNYIPTNIGGIKMFPEYSQLIPRKLY 579
JSYZ201909-1      ................................................................................................................................. 579
JSZJ202104-1      ................................................................................................................................. 579
SDQD20200424-830  ..................................................................................................................R.............. 579
CH-HN-H           ................................................................................................................................. 579
SCABTC-MS         ................................................................................................................................. 579
```

B: PPV2 capsid protein alignment

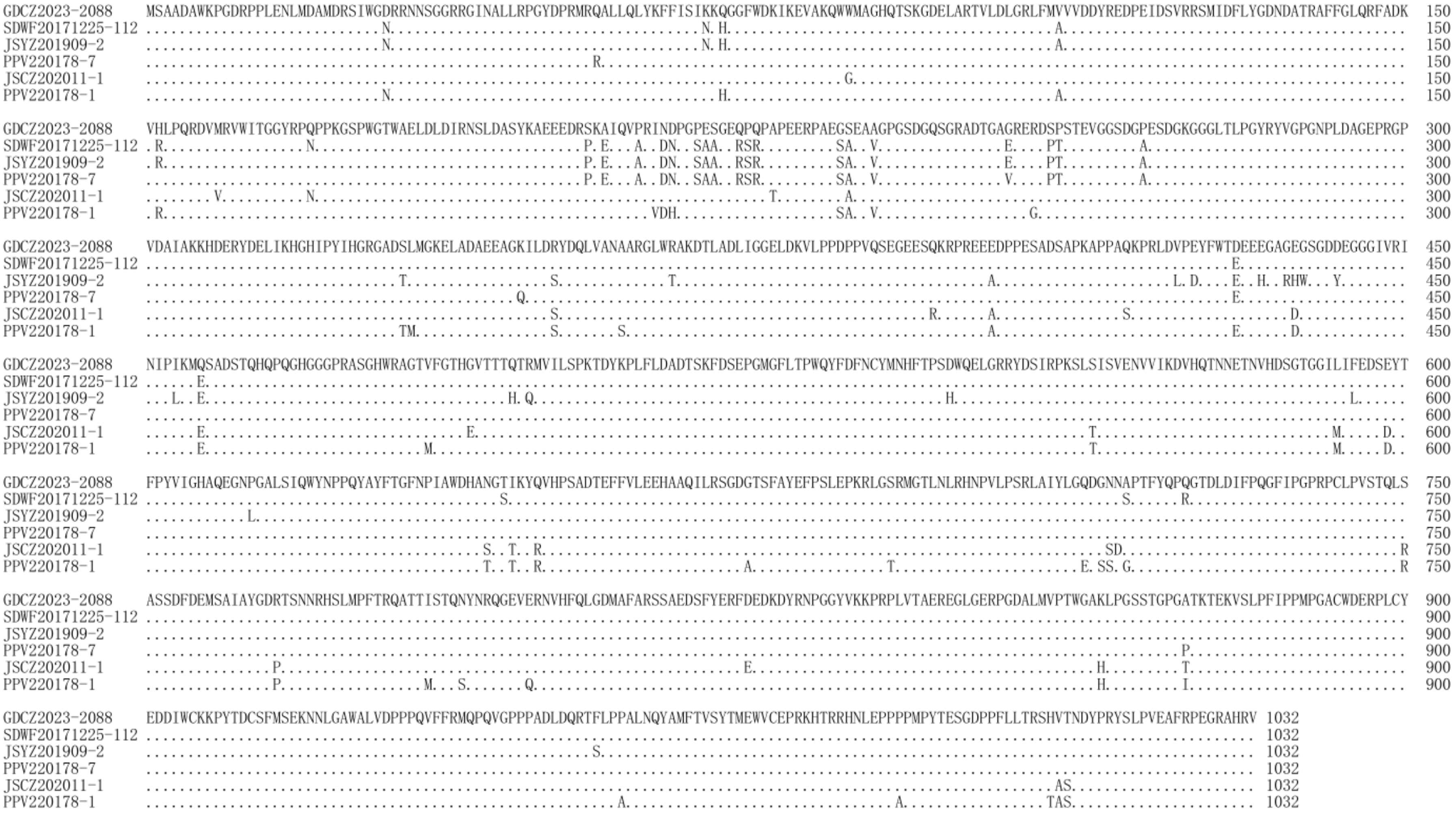

C: PPV3 capsid protein alignment

```
GDCZ2023-2088    MSAADAWKPGDRPPLENLMDAMDRSIWGDRRNNSGGRRGINALLRPGYDPRMRQALLQLYKFFISIKKQGGFWDKIKEVAKQWWMAGHQTSKGDELARTVLDLGRLFMVVVDDYREDPEIDSVRRSMIDFLYGDNDATRAFFGLQRFADK  150
SDWF20171225-112 ............................N.....................................N.H.......................................A.........................................  150
JSYZ201909-2     ............................N.....................................N.H.......................................A.........................................  150
PPV220178-7      .....................................................R................................................................................................  150
JSCZ202011-1     ...................................................................................G..................................................................  150
PPV220178-1      ............................N.......................................H.......................................A.........................................  150

GDCZ2023-2088    VHLPQRDVMRVWITGGYRPQPPKGSPWGTWAELDLDIRNSLDASYKAEEEDRSKAIQVPRINDPGPESGEQPQPAPEERPAEGSEAAGPGSDGQSGRADTGAGRERDSPSTEVGGSDGPESDGKGGGLTLPGYRYVGPGNPLDAGEPRGP  300
SDWF20171225-112 .R.................N.................................P.E..A..DN..SAA..RSR.........SA..V.................E....PT.........A...............................  300
JSYZ201909-2     .R.................N.................................P.E..A..DN..SAA..RSR.........SA..V.................E....PT.........A...............................  300
PPV220178-7      .....................................................P.E..A..DN..SAA..RSR.........SA..V.................V....PT.........A...............................  300
JSCZ202011-1     ........V..........N.......................................................T.......A..................................................................  300
PPV220178-1      .R..........................................................VDH...................SA..V..................G..........................................  300

GDCZ2023-2088    VDAIAKKHDERYDELIKHGHIPYIHGRGADSLMGKELADAEEAGKILDRYDQLVANAARGLWRAKDTLADLIGGELDKVLPPDPPVQSEGEESQKRPREEEDPPESADSAPKAPPAQKPRLDVPEYFWTDEEEGAGEGSGDDEGGGIVRI  450
SDWF20171225-112 .................................................................................................................................E....................  450
JSYZ201909-2     ..............................T.................S.............T.....................................A.....................L.D....E..H..RHW...Y........  450
PPV220178-7      ............................................Q....................................................................................E....................  450
JSCZ202011-1     ................................................S............................................R......A...............S...................D.............  450
PPV220178-1      ..............................TM................S.......S...........................................A............................E......D.............  450

GDCZ2023-2088    NIPIKMQSADSTQHQPQGHGGGPRASGHWRAGTVFGTHGVTTTQTRMVILSPKTDYKPLFLDADTSKFDSEPGMGFLTPWQYFDFNCYMNHFTPSDWQELGRRYDSIRPKSLSISVENVVIKDVHQTNNETNVHDSGTGGILIFEDSEYT  600
SDWF20171225-112 ......E...............................................................................................................................................  600
JSYZ201909-2     ...L..E....................................H.Q.................................................H...............................................L......  600
PPV220178-7      ......................................................................................................................................................  600
JSCZ202011-1     ......E...............................E.........................................................................T............................M.....D..  600
PPV220178-1      ......E..........................M...............................................................................T............................M.....D..  600

GDCZ2023-2088    FPYVIGHAQEGNPGALSIQWYNPPQYAYFTGFNPIAWDHANGTIKYQVHPSADTEFFVLEEHAAQILRSGDGTSFAYEFPSLEPKRLGSRMGTLNLRHNPVLPSRLAIYLGQDGNNAPTFYQPQGTDLDIFPQGFIPGPRPCLPVSTQLS  750
SDWF20171225-112 ..........................................S.........................................................................S......R..........................  750
JSYZ201909-2     ............L.........................................................................................................................................  750
PPV220178-7      ......................................................................................................................................................  750
JSCZ202011-1     ........................................S..T..R...................................................................SD.................................R  750
PPV220178-1      ........................................T..T..R........................A................T......................E.SS.G.................................R  750

GDCZ2023-2088    ASSDFDEMSAIAYGDRTSNNRHSLMPFTRQATTISTQNYNRQGEVERNVHFQLGDMAFARSSAEDSFYERFDEDKDYRNPGGYVKKPRPLVTAEREGLGERPGDALMVPTWGAKLPGSSTGPGATKTEKVSLPFIPPMPGACWDERPLCY  900
SDWF20171225-112 ......................................................................................................................................................  900
JSYZ201909-2     ......................................................................................................................................................  900
PPV220178-7      ...........................................................................................................................P..........................  900
JSCZ202011-1     ...............P.......................................................E..........................................H........T..........................  900
PPV220178-1      ...............P.................M...S.......Q.....................................................................H........I..........................  900

GDCZ2023-2088    EDDIWCKKPYTDCSFMSEKNNLGAWALVDPPPQVFFRMQPQVGPPPADLDQRTFLPPALNQYAMFTVSYTMEWVCEPRKHTRRHNLEPPPPMPYTESGDPPFLLTRSHVTNDYPRYSLPVEAFRPEGRAHRV 1032
SDWF20171225-112 .................................................................................................................................... 1032
JSYZ201909-2     .....................................................S.............................................................................. 1032
PPV220178-7      .................................................................................................................................... 1032
JSCZ202011-1     ............................................................................................................AS...................... 1032
PPV220178-1      ........................................................A................................A.................TAS...................... 1032
```

D: PPV5 capsid protein alignment

```
SCNJ2023-1865     MSFSGYSKNLPPGLEEVTFPFWVDFLLARIADFINWCGFYNIKCPEAEKVFSIGQSTQVLLKWPGAQGKENRVKNFTEAAFPYMKVPVRPDNIEWIKIHEMLHNYDRQITPQTTENDLLAAITADFDQREIIHPVTGEKWVFGKKTEAFA 150
PPV520178-1       ......................................Y............................................................................................................... 150
SDWF20170530-67   ......................................Y............................................................................................................... 150
JSYZ201610-1      ......................................Y............................................................................................................... 150
JSNJ20200426-911  ......................................Y.K............................................................................................................. 150
PPV520178-2       ......................................Y............................................................................................................... 150

SCNJ2023-1865     TDLEEAVDEEDPDTEKKQPTDKTQSNNKKGEIGEKKEEGDTPTSNEEHHQSRKLLEHDSSEEQPEEAGHREQKELEDNIEDIKHGAGEDQTGTGINWPGHRYTGPGNPLPHGAPRNEIDLSAAKHDIRYKQYSRYGHWPYIWAPYIDKKM 300
PPV520178-1       ...................................................................................................................................................... 300
SDWF20170530-67   ...................................................................................................................................................... 300
JSYZ201610-1      .......E........................S..................................................................................................................... 300
JSNJ20200426-911  .....T.....................E...V...................................................................................................................... 300
PPV520178-2       .........................D............................................................................................................................ 300

SCNJ2023-1865     QEDIREIVKKGLGLEGKLLGNLISALWQAKYRLGAPIYEILKTILPPKSMPTKESVEKHLPKPLPIDPPQTSLPGASPPRTPDLGGETGMNEEPPAKRRMTEDRCDSTTRCETLDTQYEDSKMAGGGGGGGNQPKSSWIGGAFFTDTTVT 450
PPV520178-1       ...................................................................................................................................................... 450
SDWF20170530-67   ...................................................................................................................................................... 450
JSYZ201610-1      ........R............................................................................................................................................. 450
JSNJ20200426-911  ...................................................................................................................................................... 450
PPV520178-2       ..E..............................................................................L............................................EMCIRDR................. 450

SCNJ2023-1865     TYGTRRCVLSSFPHNYCTTESGDHIPSLVVCTPWYYYDLNILSAHFSPSAWQTLLEEYDAFKPLKLEVKIKEIVVKDVNNMTGKQCCDTVSDNAMAAVLCFEDTHYELPYVLGGGQLTVPGHLPGQTYELPKYCYRTVGKPHSEMWSPVD 600
PPV520178-1       ...................................................................................................................................................... 600
SDWF20170530-67   ...................................................................................................................................................... 600
JSYZ201610-1      ...................................................................................................................................................... 600
JSNJ20200426-911  ....................................................................................................................................................I. 600
PPV520178-2       ....................................................................................................................................................I. 600

SCNJ2023-1865     GSKRAHLDMPFVQPTQNTEFFILENRHSTILHTGNEFFQTYDFPDLHFEQLTQYMWDARRLDNPMKGQRIQVMKNKPTENKDQMFGIRASSYLVPWIVNSLNRPAMFLQGGRLKDGDYSIVGPGTREQATYHYFNDTPVVVERDIYKFTT 750
PPV520178-1       ...................................................................................................................................................... 750
SDWF20170530-67   ...................................................................................................................................................... 750
JSYZ201610-1      ...................................................................................................................................................... 750
JSNJ20200426-911  ...................................................................................................................................................... 750
PPV520178-2       ...................................................................................................................................................... 750

SCNJ2023-1865     SMLKRETQQPGPRTQETTVKTPDGTIIITTNSLAYGQVPENIDNIPSDHKAAFGVTGYRLAVAEQRGYSTPGMPSHIREILLTKTPKLLEKDQQEITFPNFEGSVSEKTSANLESQIWAYIPNTDNKHNCGTPPLSIWGMENPPPMVFLR 900
PPV520178-1       ...................................................................................................................................................... 900
SDWF20170530-67   ...................................................................................................................................................... 900
JSYZ201610-1      ...................................................................................................................................................... 900
JSNJ20200426-911  ...................................................................................................................................................... 900
PPV520178-2       ...................................................................................................................................................... 900

SCNJ2023-1865     LLPQLGPPEKSSCSGSKPSKKFLNQYCQFLLEYTVTWAVVRRKKHTPRWNPMPGVTIPTYNNDPVYILDQNGFYKLPETVWTAKQRVRARR 991
PPV520178-1       ........................................................................................... 991
SDWF20170530-67   ........................................................................................... 991
JSYZ201610-1      ........................................................................................... 991
JSNJ20200426-911  ........................................................................................... 991
PPV520178-2       ........................................................................................... 991
```

E: PPV6 capsid protein alignment

```
GDCZ2023-2439    MSRSTQRDLWSLLKERLETYKDRVKDYGILVPERPSTSASYFSRDPPPDPPNVKFDKPYQDVDRFWFPTDDYTDWYVWHGEDRPPRFTEHSGVQGFKTRCEGRLPLMSSDPKFCPKSDFWDQFANYDEGSPSPQIGESVSWSDHFGSPDP  150
PPV620178-1      .............R...........Y.................K.......T..................................................G....P.......TQN.......FN.......................  150
JSTZ20181121-431 .............R...........Y.................K.......T.....A............................................G....P.......RQN.......FN.......................  150
SDWF20170530-68  ....S........R....R......Y...........L.....K.......T..................................................G....P.......LQN.......F......T.................  150
FJ2017           .............R...........Y.................K.......T..................................................G....P.......IQN.......F........................  150
BJ               ......................................................................................................G...............................................  150

GDCZ2023-2439    SHHQEVRDADEVVSERQQYKNRIVTLLRKVYWAKQWSGKLQINVPSLESLYEQIPYMLAYMDADNWRQNLLAAKTLRTTLEAFSCVPDPSTCDVTISTPLSGETDPASFAKYLCSLVCNRSQEKEQAQTPSLSPSKQEGQMSSPDSASIS  300
PPV620178-1      ......................................................................................................................................................  300
JSTZ20181121-431 ......................................................................................................................................................  300
SDWF20170530-68  ............T.........................................................................................................................................  300
FJ2017           ......................................................................................................................................................  300
BJ               ......................................................................................................................................................  300

GDCZ2023-2439    QPPPESHKDRLLPKTDPLQEAGPIPAPPTVQKPIIPKGAGGGGSSGFIIPPKPPSPDHTPDPPPPPPPSPIPPPTSAPDAEEHELERAKQEKQEEDELMQRIKSGEGEGERGGFVLPSHHYTGPRNPVPAGKPADPVDESSARHDIRYGQ  450
PPV620178-1      .......................L.....A.....S.......................K..........................................................................................  450
JSTZ20181121-431 .......................L.....A.....S.......................K..........................................................................................  450
SDWF20170530-68  .......................L.....A.....S.......................K..........................................................................................  450
FJ2017           .......................L.....A.....S.......................K..........................................................................................  450
BJ               ......................T............S..................................................................................................................  450

GDCZ2023-2439    RLKHGDWPYLWGKDLDNAQRDEIIKALHSHVKVGTQLAGNIVRSIWKAKELLTEPVYELLKSILPPSDLSKVPLPHSQQTDRTEDPETPGETRGTGSDSPRSPRPSGSTEDGGGPSSESRLPGTKVPVDPSATTSEAKRQRTEEGMDISS  600
PPV620178-1      ......................................................................................................................................................  600
JSTZ20181121-431 ......................................................................................................................................................  600
SDWF20170530-68  ......................................................................................................................................................  600
FJ2017           ......................................................................................................................................................  600
BJ               ......................................................................................................................................................  600

GDCZ2023-2439    CCPGGISASGAASNNSGLACGGGGGTNLGTESLVSGCQFGKNSVITSSFRRCLISPWPDKYCCSSAHDLIPGVVYETPWCYYDLNVISAHFSPSAWQRLLEDYDAFRPKSLKVTIQSLVFKDVCQGAEKQTTVQDSQSATIAIFEDKDYD  750
PPV620178-1      ......................................................................................................................................................  750
JSTZ20181121-431 ......................................................................................................................................................  750
SDWF20170530-68  ......................................................................................................................................................  750
FJ2017           ......................................................................................................................................................  750
BJ               ......................................................................................................................................................  750

GDCZ2023-2439    YPYEMGGGQKTVPGHLPGQPYNLPKYSYRTLGSVKESIMAHHGGSGYTFKSNQDTELFLLETHDSTLIRGGATFEQYYEFPNDLPFENLTQYPWDIRRQDNPLYQQRITVMSGSDRDQAGILGGDLYSPFRFKGHDRPAMWLPGQRLIQG  900
PPV620178-1      ...V.................................NR.SM......................A......G..............................................V...D..F........................  900
JSTZ20181121-431 ...V.................................NR.SM......................A......G..............................................V...D..F........................  900
SDWF20170530-68  ...V.................................NR.SM......................A......G..............................................V...D..F........................  900
FJ2017           ...V.................................NR.SM......................A......G..................................................D..F........................  900
BJ               ...V.................................S.................................G.....................................................F........................  900

GDCZ2023-2439    KFIDTHPIPNTGRSGVHPSDFHTRGDGHGDTHRTHEEKIYTLDTGLAAMPRAAHRPTLQPGPRTLSHAVRRPDGSTVVTANACAYAYTQENPHQEPWSDLNVRHTMYRLAYQHQKGFQQPGDPLHIRTHACYGDGDVNIPKDESLWPTVL 1050
PPV620178-1      ..................N..................R..S.......................................................................R........................T...E........ 1050
JSTZ20181121-431 ..................N..................R..S.......................................................................R........................T...E........ 1050
SDWF20170530-68  ..................N..................R..S.......................................................................R........................T...E........ 1050
FJ2017           ..................N..................R..S.......................................................................R........................T...E........ 1050
BJ               ...................................................................................................................................................... 1050

GDCZ2023-2439    GSCPEKSPACLESQIWCKTPNVDMVYGEHTPPLALWGMRAPPPHVFLRMLVQEGPPNVSTWIPDRSGPTFINLYGQFLLCLTMVWEVKPRPKSIKQWNPRPPISIPVGQSGPAFILDQNGYYRLPEHVWTARERIRNKR 1189
PPV620178-1      ...T..................................H...........A.........CR.AQ..Q....Q.......F.....................................D..........S......S.. 1189
JSTZ20181121-431 ...T..................................H...........A.........CR.AQ..Q....Q.......F.....................................D..........S......S.. 1189
SDWF20170530-68  ...T..................................H...........A.........CR.AQ..Q....Q.......F.....................................D..........S......S.. 1189
FJ2017           ...T..................................H...........A.........CR.AQ..Q....Q.......F.....................................D..........S......S.. 1189
BJ               ...T..............................................A.........CR.AQ..Q....Q.......F.....................................D..........S......S.. 1189
```

F: PPV7 capsid protein alignment

```
HNZMD2023-1903    MAEHITLSNTFMAYWENDPYQYPSYTPFQKNKVLSYNTGWHILPNILWRHFLSPKQWYELCINYEAYHVEGTSTTVFNPIPITNNLAIQGTNTFTAFNNTIYSLGTTDDLYETGYHNWYEDELWRSWYVAYKEGLVPKRNVPTKDGVGNS 150
18FJZZ35          ...............................N.............V.............................I.............................F......S..........................D....E..... 150
HBTS20180519-152  ...............................N...........L.V...........................................................F......S..........................D....E..... 150
GX2               ...............................N.............V...........................................................F......S..........................D....E..... 150
JSCZ201710-1      .........................K...R.E.............V.............................I.............................A.................................DI...E..... 150
AHbz              ...............................E....S................................................................................................................. 150

HNZMD2023-1903    WNRLLLPIYRWSAPITAPETNWTWVWNTSKGTGAQNYPTAGTTWPHTDSGTEQVAAPAGCFWDPFTNPDSIQELRPGKNAMSFHWKTHGADESCWYNLDSLVKLFPYTPESGYSHNIKNKKYIGPPGSRIVNDNFHHPSLQTSFSSENIK 300
18FJZZ35          ....T..K.L..................G...............................................................H........................RD...Q....T.V..E..Q...P.....T..L. 300
HBTS20180519-152  ....T..K.L..................G...............................................................H........................RD...Q....T.V..E..Q...P.....T..L. 300
GX2               .......K.L..................G...............................................................H........................RD...Q....T.V..E..Q...P.....T..L. 300
JSCZ201710-1      .......K.L..................G...............................................................H........................Q....K.........E..Q...P.....T..L. 300
AHbz              ............................G..E...A........................................................H......................S.Q....K.........E..Q...P.....T..L. 300

HNZMD2023-1903    VFIEHEVPNVLNAPIVPIQWFWIELERNLIEDKKTEKPQIGWPGTEWATPKYPPMNNFIKGIPLTDENGTLVKTVTMGCFRNSIHLSVKKRRSRMFAPTWGPMSVEMTHGIDSPFVLPTVRYRTGGARRSWQAKTRDGRDQQPQQPWYQW 450
18FJZZ35          .....D............................V....L...........................H...........I...L...C.........................A...................R...TK...T....... 450
HBTS20180519-152  .....D............................V....L...........................H...........I...L...C.........................A...................R...TK...T....... 450
GX2               .....D............................V....L...........................H...........I...L...C.........................A...................R...TK...T....... 450
JSCZ201710-1      .....D............................I....L...........................................L...C.........................A...................R...V....Q....... 450
AHbz              .....D......T.....................I....L.....................V.........................C.........................A...................R...V...HQHHS.... 450

HNZMD2023-1903    NPYMTGTYTSTTTTSTYTTTTSRK 474
18FJZZ35          ...G.................... 474
HBTS20180519-152  ...G.................... 474
GX2               ...G.................... 474
JSCZ201710-1      ...E.................... 474
AHbz              ...G.................... 474
```
