# Supplementary material for: Positivity Status and Molecular Characterization of Porcine Parvoviruses 1 Through 8 (PPV1-PPV8) from Slaughtered Pigs in China
Source: Animals (Basel). 2024 Nov 12;14(22):3238. doi: 10.3390/ani14223238 (PMC11591526; doi:10.3390/ani14223238)
Supplement: Supplementary file 1 [file animals-14-03238-s001.zip › Table S1 Clinical sample infection status R1.pdf]

Table S1. Clinical sample infection status

Sample	Type	Region	PPV1	PPV2	PPV3	PPV4	PPV5	PPV6	PPV7	PPV8
1640	Lung	Shuangyashan, Heilongjiang								
1641	Lung	Shuangyashan, Heilongjiang								
1642	Lung	Shuangyashan, Heilongjiang								
1643	Lung	Shuangyashan, Heilongjiang								
1644	Lung	Shuangyashan, Heilongjiang							+	
1645	Lung	Shuangyashan, Heilongjiang								
1646	Lung	Shuangyashan, Heilongjiang								
1647	Lung	Shuangyashan, Heilongjiang								
1648	Lung	Shuangyashan, Heilongjiang								
1649	Lung	Shuangyashan, Heilongjiang	+							
1650	Lung	Shuangyashan, Heilongjiang			+				+	
1651	Lung	Shuangyashan, Heilongjiang							+	
1652	Lung	Shuangyashan, Heilongjiang							+	
1653	Lung	Shuangyashan, Heilongjiang							+	
1654	Lung	Shuangyashan, Heilongjiang			+					
1655	Lung	Shuangyashan, Heilongjiang							+	
1656	Lung	Shuangyashan, Heilongjiang						+	+	
1657	Lung	Shuangyashan, Heilongjiang			+					
1658	Lung	Shuangyashan, Heilongjiang							+	
1659	Lung	Shuangyashan, Heilongjiang							+	
1660	Lung	Shuangyashan, Heilongjiang								
1661	Lung	Shuangyashan, Heilongjiang								
1662	Lung	Shuangyashan, Heilongjiang								
1663	Lung	Shuangyashan, Heilongjiang			+					
1664	Lung	Shuangyashan, Heilongjiang								
1665	Lung	Shuangyashan, Heilongjiang			+				+	
1666	Lung	Shuangyashan, Heilongjiang								
1667	Lung	Shuangyashan, Heilongjiang								
1668	Lung	Shuangyashan, Heilongjiang		+	+				+	
1669	Lung	Shuangyashan, Heilongjiang							+	
1670	Lung	Shuangyashan, Heilongjiang							+	
1733	Lung	Linyi, Shandong	+							

1734	Lung	Linyi, Shandong							+
1735	Lung	Linyi, Shandong		+					
1736	Lung	Linyi, Shandong							
1737	Lung	Linyi, Shandong							+
1738	Lung	Linyi, Shandong		+	+				
1739	Lung	Linyi, Shandong			+				
1740	Lung	Linyi, Shandong			+				
1741	Lung	Linyi, Shandong							
1742	Lung	Linyi, Shandong							
1743	Lung	Linyi, Shandong							
1744	Lung	Linyi, Shandong							
1745	Lung	Zibo, Shandong							
1746	Lung	Zibo, Shandong	+						
1747	Lung	Zibo, Shandong	+						
1748	Lung	Zibo, Shandong	+						
1749	Lung	Zibo, Shandong	+		+		+		
1750	Lung	Zibo, Shandong	+	+	+				
1751	Lung	Zibo, Shandong							+
1752	Lung	Zibo, Shandong		+					
1753	Lung	Zibo, Shandong		+	+				+
1754	Lung	Zibo, Shandong	+		+				
1755	Lung	Zibo, Shandong							
1756	Lung	Zibo, Shandong			+				+
1757	Lung	Zibo, Shandong			+			+	+
1758	Lung	Zibo, Shandong	+	+	+			+	+
1759	Lung	Zibo, Shandong		+					
1760	Lung	Zibo, Shandong	+	+	+				
1761	Lung	Zibo, Shandong	+	+					
1783	Lung	Shantou, Guangdong	+						
1784	Lung	Shantou, Guangdong	+						
1785	Lung	Shantou, Guangdong	+		+				
1786	Lung	Shantou, Guangdong	+				+		+
1787	Lung	Shantou, Guangdong	+						+
1788	Lung	Shantou, Guangdong							

1789	Lung	Shantou, Guangdong	+						
1790	Lung	Shantou, Guangdong	+						
1791	Lung	Shantou, Guangdong	+	+					
1792	Lung	Shantou, Guangdong		+	+				+
1793	Lung	Shantou, Guangdong							
1794	Lung	Shantou, Guangdong							
1795	Lung	Shantou, Guangdong							
1796	Lung	Shantou, Guangdong							
1797	Lung	Shantou, Guangdong			+				
1798	Lung	Shantou, Guangdong		+					
1799	Lung	Shantou, Guangdong			+				
1800	Lung	Shantou, Guangdong							
1801	Lung	Shantou, Guangdong			+				
1802	Lung	Shantou, Guangdong							
1803	Lung	Shantou, Guangdong			+				
1804	Lung	Shantou, Guangdong							+
1805	Lung	Shantou, Guangdong							
1806	Lung	Shantou, Guangdong			+				+
1807	Lung	Shantou, Guangdong			+				
1808	Lung	Shantou, Guangdong		+	+		+		
1809	Lung	Shantou, Guangdong	+		+				
1810	Lung	Shantou, Guangdong						+	
1811	Lung	Shantou, Guangdong		+			+		+
1812	Lung	Shantou, Guangdong			+			+	
1845	Lung	Dezhou, Shandong			+				
1846	Lung	Dezhou, Shandong			+				
1847	Lung	Dezhou, Shandong			+			+	
1848	Lung	Dezhou, Shandong			+				
1849	Lung	Dezhou, Shandong	+		+				
1850	Lung	Dezhou, Shandong			+				
1851	Lung	Dezhou, Shandong							
1852	Lung	Dezhou, Shandong			+				+
1853	Lung	Neijiang, Sichuan		+					
1854	Lung	Neijiang, Sichuan					+		+

1855	Lung	Neijiang, Sichuan						+
1856	Lung	Neijiang, Sichuan	+					+
1857	Lung	Neijiang, Sichuan						+
1858	Lung	Neijiang, Sichuan						+
1859	Lung	Neijiang, Sichuan		+			+	+
1860	Lung	Neijiang, Sichuan						+
1861	Lung	Neijiang, Sichuan						+
1862	Lung	Neijiang, Sichuan	+					+
1863	Lung	Neijiang, Sichuan						
1864	Lung	Neijiang, Sichuan						+
1865	Lung	Neijiang, Sichuan				+		+
1866	Lung	Neijiang, Sichuan				+		+
1867	Lung	Neijiang, Sichuan				+		+
1868	Lung	Neijiang, Sichuan	+					+
1869	Lung	Neijiang, Sichuan		+				+
1870	Lung	Neijiang, Sichuan						+
1871	Lung	Neijiang, Sichuan						+
1872	Lung	Neijiang, Sichuan	+					+
1873	Lung	Neijiang, Sichuan	+					
1874	Lung	Neijiang, Sichuan						+
1875	Lung	Neijiang, Sichuan						
1876	Lung	Neijiang, Sichuan						
1877	Lung	Neijiang, Sichuan						+
1878	Lung	Neijiang, Sichuan						
1879	Lung	Neijiang, Sichuan	+					+
1880	Lung	Neijiang, Sichuan						
1881	Lung	Neijiang, Sichuan						
1882	Lung	Neijiang, Sichuan						
1883	Lung	Neijiang, Sichuan		+				
1884	Lung	Neijiang, Sichuan						
1885	Lung	Neijiang, Sichuan						
1886	Lung	Neijiang, Sichuan						
1887	Lung	Zhumadian, Henan						
1888	Lung	Zhumadian, Henan						+

1889	Lung	Zhumadian, Henan							+
1890	Lung	Zhumadian, Henan							
1891	Lung	Zhumadian, Henan							
1892	Lung	Zhumadian, Henan							+
1893	Lung	Zhumadian, Henan							
1894	Lung	Zhumadian, Henan							+
1895	Lung	Zhumadian, Henan							
1896	Lung	Zhumadian, Henan		+					
1897	Lung	Zhumadian, Henan							+
1898	Lung	Zhumadian, Henan		+					+
1899	Lung	Zhumadian, Henan							
1900	Lung	Zhumadian, Henan							+
1901	Lung	Zhumadian, Henan		+				+	+
1902	Lung	Zhumadian, Henan							
1903	Lung	Zhumadian, Henan			+			+	+
1904	Lung	Zhumadian, Henan							
1905	Lung	Zhumadian, Henan							
1906	Lung	Zhumadian, Henan							
1907	Lung	Zhumadian, Henan							
1908	Lung	Zhumadian, Henan	+						+
1909	Lung	Zhumadian, Henan							
1910	Lung	Zhumadian, Henan	+						+
1911	Lung	Zhumadian, Henan			+		+	+	+
1912	Lung	Zhumadian, Henan							
1913	Lung	Zhumadian, Henan							
1914	Lung	Zhumadian, Henan			+			+	+
1915	Lung	Zhumadian, Henan		+	+				+
1916	Lung	Zhumadian, Henan							+
1947	Lung	Zhoukou, Henan			+			+	
1948	Lung	Zhoukou, Henan			+			+	
1949	Lung	Zhoukou, Henan						+	
1950	Lung	Zhoukou, Henan			+				+
1951	Lung	Zhoukou, Henan		+					+
1952	Lung	Zhoukou, Henan		+					

1953	Lung	Zhoukou, Henan		+	+		+	+	+
1954	Lung	Zhoukou, Henan		+	+				
1955	Lung	Zhoukou, Henan		+	+		+		
1956	Lung	Zhoukou, Henan			+				+
1957	Lung	Zhoukou, Henan					+		
1958	Lung	Zhoukou, Henan					+	+	
1959	Lung	Zhoukou, Henan						+	+
1960	Lung	Zhoukou, Henan		+			+		+
1961	Lung	Zhoukou, Henan		+	+				
1962	Lung	Zhoukou, Henan		+	+		+	+	
1963	Lung	Zhoukou, Henan		+	+		+	+	
1964	Lung	Zhoukou, Henan							+
1965	Lung	Zhoukou, Henan			+			+	
1966	Lung	Zhoukou, Henan		+				+	+
1967	Lung	Zhoukou, Henan		+	+			+	+
1968	Lung	Zhoukou, Henan		+			+		
1969	Lung	Zhoukou, Henan	+	+	+		+	+	+
1970	Lung	Zhoukou, Henan		+	+				
1971	Lung	Zhoukou, Henan		+	+			+	+
1972	Lung	Zhoukou, Henan		+	+			+	
1973	Lung	Zhoukou, Henan		+	+			+	+
1974	Lung	Zhoukou, Henan		+	+			+	
1975	Lung	Zhoukou, Henan		+	+			+	
1976	Lung	Zhoukou, Henan		+			+	+	
1977	Lung	Zhoukou, Henan		+			+		
1978	Lung	Zhoukou, Henan		+	+				
1979	Lung	Zhoukou, Henan		+	+				
1980	Lung	Zhoukou, Henan			+				
1981	Lung	Zhoukou, Henan		+	+				
1982	Lung	Zhoukou, Henan		+					
1983	Lung	Zhoukou, Henan		+	+				
1984	Lung	Zhoukou, Henan		+	+				
1985	Lung	Zhoukou, Henan		+	+				
1986	Lung	Zhoukou, Henan		+	+				

2077	Lung	Chaozhou,Guangdong		+	+				
2078	Lung	Chaozhou,Guangdong		+					
2079	Lung	Chaozhou,Guangdong		+	+				+
2080	Lung	Chaozhou,Guangdong		+	+				+
2081	Lung	Chaozhou,Guangdong		+					
2082	Lung	Chaozhou,Guangdong		+					
2083	Lung	Chaozhou,Guangdong		+	+			+	
2084	Lung	Chaozhou,Guangdong		+					+
2085	Lung	Chaozhou,Guangdong		+	+			+	+
2086	Lung	Chaozhou,Guangdong		+	+				
2087	Lung	Chaozhou,Guangdong			+				
2088	Lung	Chaozhou,Guangdong		+	+				+
2089	Lung	Chaozhou,Guangdong		+	+				
2090	Lung	Chaozhou,Guangdong		+					
2091	Lung	Chaozhou,Guangdong		+	+				+
2092	Lung	Chaozhou,Guangdong		+	+				
2093	Lung	Chaozhou,Guangdong		+	+				+
2094	Lung	Chaozhou,Guangdong		+					+
2095	Lung	Chaozhou,Guangdong		+	+				+
2096	Lung	Chaozhou,Guangdong		+	+				+
2133	Lung	Chaozhou,Guangdong		+					
2134	Lung	Chaozhou,Guangdong		+					+
2135	Lung	Chaozhou,Guangdong	+	+	+				+
2136	Lung	Chaozhou,Guangdong							+
2137	Lung	Chaozhou,Guangdong		+	+				
2138	Lung	Chaozhou,Guangdong		+	+				
2139	Lung	Chaozhou,Guangdong		+	+				+
2140	Lung	Chaozhou,Guangdong		+	+				
2141	Lung	Chaozhou,Guangdong		+	+				
2142	Lung	Chaozhou,Guangdong		+	+				
2143	Lung	Chaozhou,Guangdong							
2144	Lung	Chaozhou,Guangdong			+				+
2145	Lung	Chaozhou,Guangdong		+	+				
2146	Lung	Chaozhou,Guangdong		+	+				

2147	Lung	Chaozhou,Guangdong			+				+
2148	Lung	Chaozhou,Guangdong		+	+				+
2149	Lung	Chaozhou,Guangdong		+	+				
2150	Lung	Chaozhou,Guangdong		+	+				+
2151	Lung	Chaozhou,Guangdong		+	+				
2152	Lung	Chaozhou,Guangdong		+	+				+
2153	Lung	Chaozhou,Guangdong		+	+				
2154	Lung	Chaozhou,Guangdong		+	+				
2376	Lung	Chaozhou,Guangdong							
2377	Lung	Chaozhou,Guangdong	+						
2378	Lung	Chaozhou,Guangdong							
2379	Lung	Chaozhou,Guangdong							
2380	Lung	Chaozhou,Guangdong			+				
2381	Lung	Chaozhou,Guangdong							
2382	Lung	Chaozhou,Guangdong							
2383	Lung	Chaozhou,Guangdong		+					
2384	Lung	Chaozhou,Guangdong							
2385	Lung	Chaozhou,Guangdong							
2386	Lung	Chaozhou,Guangdong		+					
2387	Lung	Chaozhou,Guangdong							
2388	Lung	Chaozhou,Guangdong			+				
2389	Lung	Chaozhou,Guangdong							
2390	Lung	Chaozhou,Guangdong							
2391	Lung	Chaozhou,Guangdong							
2392	Lung	Chaozhou,Guangdong							
2393	Lung	Chaozhou,Guangdong							
2394	Lung	Chaozhou,Guangdong							
2395	Lung	Chaozhou,Guangdong							
2437	Lung	Chaozhou,Guangdong			+			+	+
2438	Lung	Chaozhou,Guangdong			+				
2439	Lung	Chaozhou,Guangdong			+			+	
2440	Lung	Chaozhou,Guangdong	+		+			+	
2441	Lung	Chaozhou,Guangdong			+				
2442	Lung	Chaozhou,Guangdong		+	+				

2443	Lung	Chaozhou,Guangdong							
2444	Lung	Chaozhou,Guangdong			+				
2445	Lung	Chaozhou,Guangdong	+		+				
2446	Lung	Chaozhou,Guangdong	+		+				
2447	Lung	Chaozhou,Guangdong	+		+				+
2448	Lung	Chaozhou,Guangdong	+		+				
2449	Lung	Chaozhou,Guangdong	+						
2450	Lung	Chaozhou,Guangdong	+	+	+				
2451	Lung	Chaozhou,Guangdong			+				
2452	Lung	Chaozhou,Guangdong	+		+				
2453	Lung	Chaozhou,Guangdong	+		+				
2454	Lung	Chaozhou,Guangdong	+	+					
2455	Lung	Chaozhou,Guangdong			+				+
2456	Lung	Chaozhou,Guangdong			+				
2531	Lung	Chaozhou,Guangdong			+				+
2532	Lung	Chaozhou,Guangdong		+	+				
2533	Lung	Chaozhou,Guangdong		+	+				+
2534	Lung	Chaozhou,Guangdong		+	+				
2535	Lung	Chaozhou,Guangdong		+	+				+
2536	Lung	Chaozhou,Guangdong		+	+				+
2537	Lung	Chaozhou,Guangdong			+				
2538	Lung	Chaozhou,Guangdong		+	+				
2539	Lung	Chaozhou,Guangdong		+	+				
2540	Lung	Chaozhou,Guangdong			+				
2541	Lung	Chaozhou,Guangdong			+				
2542	Lung	Chaozhou,Guangdong			+				+
2543	Lung	Chaozhou,Guangdong			+				+
2544	Lung	Chaozhou,Guangdong			+				+
2545	Lung	Chaozhou,Guangdong			+				+
2546	Lung	Chaozhou,Guangdong		+				+	
2547	Lung	Chaozhou,Guangdong			+				
2548	Lung	Chaozhou,Guangdong		+	+				
2549	Lung	Chaozhou,Guangdong							
2550	Lung	Chaozhou,Guangdong			+				+

2551	Lymph node	Chaozhou,Guangdong		+	+			+
2552	Lymph node	Chaozhou,Guangdong		+	+			+
2553	Lymph node	Chaozhou,Guangdong		+	+			+
2554	Lymph node	Chaozhou,Guangdong		+	+			+
2555	Lymph node	Chaozhou,Guangdong			+			+
2556	Lymph node	Chaozhou,Guangdong		+	+			+
2557	Lymph node	Chaozhou,Guangdong	+	+	+	+	+	+
2558	Lymph node	Chaozhou,Guangdong		+	+			+
2559	Lymph node	Chaozhou,Guangdong	+	+	+	+	+	+
2560	Lymph node	Chaozhou,Guangdong		+	+		+	+
2561	Lymph node	Chaozhou,Guangdong		+	+			+
2562	Lymph node	Chaozhou,Guangdong		+	+			+
2563	Lymph node	Chaozhou,Guangdong		+	+			+
2564	Lymph node	Chaozhou,Guangdong		+	+			+
2565	Lymph node	Chaozhou,Guangdong		+	+			
2611	Lung	Chaozhou,Guangdong		+	+			+
2612	Lung	Chaozhou,Guangdong						
2613	Lung	Chaozhou,Guangdong		+				+
2614	Lung	Chaozhou,Guangdong	+	+	+			+
2615	Lung	Chaozhou,Guangdong	+	+	+		+	+
2616	Lung	Chaozhou,Guangdong		+	+			+
2617	Lung	Chaozhou,Guangdong		+				+
2618	Lung	Chaozhou,Guangdong	+	+				+
2619	Lung	Chaozhou,Guangdong			+			+
2620	Lung	Chaozhou,Guangdong	+	+	+			+
2621	Lung	Chaozhou,Guangdong		+				+
2622	Lung	Chaozhou,Guangdong	+	+	+			
2623	Lung	Chaozhou,Guangdong		+				+
2624	Lung	Chaozhou,Guangdong	+	+				+
2625	Lung	Chaozhou,Guangdong	+				+	
2626	Lung	Chaozhou,Guangdong		+	+			+
2627	Lung	Chaozhou,Guangdong	+	+	+		+	+
2628	Lung	Chaozhou,Guangdong		+	+			+
2629	Lung	Chaozhou,Guangdong		+				+

2630	Lung	Chaozhou,Guangdong		+					+	
2651	Lung	Beijing		+	+		+		+	
2652	Lung	Beijing		+	+					
2653	Lung	Beijing		+	+					
2654	Lung	Beijing		+	+		+		+	
2655	Lung	Beijing		+	+		+		+	
2656	Lung	Beijing		+	+			+	+	
2657	Lung	Beijing		+	+			+	+	
2658	Lung	Beijing		+	+		+	+	+	
2659	Lung	Beijing		+	+		+	+	+	
2660	Lung	Beijing	+	+	+			+	+	
2661	Lung	Beijing		+	+		+	+	+	
2662	Lung	Beijing							+	
2663	Lung	Beijing		+	+		+		+	
2664	Lung	Beijing		+	+				+	
353			47	150	176	0	29	47	149	0
